# Progesterone Hypersensitivity in Assisted Reproductive Technologies: Implications for Safety and Efficacy

**DOI:** 10.3390/jpm14010079

**Published:** 2024-01-10

**Authors:** Florica Sandru, Mihai Cristian Dumitrascu, Aida Petca, Razvan-Cosmin Petca, Alexandra-Maria Roman

**Affiliations:** 1 Department of Dermatovenerology, “Carol Davila” University of Medicine and Pharmacy, 020021 Bucharest, Romania; florica.sandru@umfcd.ro; 2 Dermatology Department, “Elias” University Emergency Hospital, 011461 Bucharest, Romania; alexandra-maria.roman@rez.umfcd.ro; 3 Department of Obstetrics and Gynecology, “Carol Davila” University of Medicine and Pharmacy, 020021 Bucharest, Romania; 4 Department of Obstetrics and Gynecology, University Emergency Hospital of Bucharest, 050098 Bucharest, Romania; 5 Department of Obstetrics and Gynecology, Elias Emergency University Hospital, 011461 Bucharest, Romania; 6 Department of Urology, “Carol Davila” University of Medicine and Pharmacy, 020021 Bucharest, Romania; 7 Department of Urology, “Prof. Dr. Th. Burghele” Clinical Hospital, 050659 Bucharest, Romania

**Keywords:** autoimmune progesterone dermatitis, progesterone hypersensitivity, in vitro fertilization, progesterone desensitization, assisted reproduction

## Abstract

The global rise in the age of childbirth, influenced by changing sociodemographic patterns, has had a notable impact on fertility rates. Simultaneously, assisted reproductive techniques (ARTs) have become increasingly prevalent due to advancements in reproductive medicine. The paper explores the intersection between the surge in ARTs and the rising number of iatrogenic autoimmune progesterone dermatitis (APD). Autoimmune progesterone dermatitis, commonly known as progesterone hypersensitivity, manifests itself as a mucocutaneous hypersensitivity syndrome. It is characterized by a wide range of dermatological symptoms, with urticaria and maculopapular rashes being the most prominent signs. Concurrently, systemic symptoms, such as fever, angioedema, and, in severe instances, anaphylaxis, may ensue. This dermatologic condition poses a significant challenge to women of childbearing age. This intricate syndrome frequently manifests itself in conjunction with menstruation or pregnancy as a reaction to physiological fluctuations in endogenous progesterone. However, given that exposure to exogenous progesterone is an integral component of various modern therapies, secondary APD has also been described. Our findings unveil a heightened likelihood of developing secondary progesterone hypersensitivity in ART patients that is attributed to the administration of exogenous progesterone through intramuscular, intravaginal, and oral routes. The study also explores available therapeutic interventions for facilitating viable pregnancies in individuals grappling with autoimmune progesterone dermatitis within the context of ARTs. This comprehensive analysis contributes valuable insights into the intricate relationship between reproductive technologies, dermatological challenges, and successful pregnancy outcomes.

## 1. Introduction

Involuntary infertility is exceedingly frequent in the general population, indicating that around one in four to five couples do not achieve a detected pregnancy after 12 months of unprotected intercourse following the cessation of contraceptive use. Additionally, approximately one in ten couples may remain unsuccessful in conceiving after two years of unprotected intercourse [1]. Age stands out as the foremost factor in female infertility, primarily attributed to the decline in ovarian reserve that comes with advancing years [2]. This underscores the need for the more frequent deployment of assisted reproduction techniques (ARTs) as there have been discernible upward trends in both average maternal age and the proportionality of advanced maternal age in recent years [3]. While a need for caution is warranted due to the association of ARTs with an increased risk in maternal and fetal health issues, their prevalence is steadily on the rise [4,5,6]. It is anticipated that around 400 million individuals, making up 3% of the world’s population, might have come into existence through the utilization of in vitro fertilization (IVF) and other fertility treatments by the year 2100; hence, more and more women are consistently subjected to elevated doses of exogenous hormones, which are a potential trigger of cutaneous hypersensitivity [7].

Autoimmune progesterone dermatitis (APD), also referred to as progesterone hypersensitivity, is a poorly recognized mucocutaneous hypersensitivity syndrome associated with an elevation in progesterone levels. Thus, this condition tends to occur in fertile women, with a mean age of 27.3 years, and potentially manifests itself during the luteal phase of the menstrual cycle, pregnancy, or post-partum period [8]. Due to APD being an under-recognized hypersensitivity reaction, the medical literature includes only about 200 reported cases of progesterone hypersensitivity to date [9]. 

The existing literature predominantly documents cases of cyclic APD-associated endogenous progesterone [10,11]. However, more and more local and systemic reactions linked to exogenous exposure have been reported, indicating a potentially isolated form of hypersensitivity to synthetic progesterone. Some reports also advocate that APD is strongly linked to the prior use of exogenous progestogens, particularly oral contraceptives (OCs) and fertility treatments [12,13,14]. Aghazadeh et al. reported that 64.3% of the cases they had studied had a history of exogenous progesterone exposure in the form of OCs or hormonal IUDs. On the other hand, the same study underlined that 92.9% of their patients had a previous history of pregnancy, which also involved a constantly elevated endogenous progesterone level [15]. As ARTs are a more recent development in common medical practice, the number of reports of patients with progesterone hypersensitivity who have also undergone such procedures is relatively small. However, an investigation into the use of such methods is crucial due to the significance of comprehending their potential future clinical implications.

In-depth accounts of catamenial dermatoses, which encompass conditions that occur in conjunction with the menstrual cycle like autoimmune progesterone or estrogen dermatitis (APD), have been thoroughly outlined [16]. The same can be said for common pregnancy or skin issues, which can overlap, to a certain degree, with the pathologies mentioned above [17,18,19]. However, dermatological phenomena in individuals undergoing assisted reproductive techniques (ARTs) remain comparatively under-explored in the realm of research. The primary objective of this narrative review is to assess the risk of progesterone hypersensitivity (APD) development in women undergoing ARTs. Additionally, the study aims to explore therapeutic options for these patients and investigate the impact of APD on fertility outcomes.

## 2. Materials and Methods

Methodical research on English-language publications from 2000 to 2023 was conducted using PubMed. The search incorporated the following specific keywords: “autoimmune progesterone dermatitis” (or “progesterone urticaria”, “progesterone dermatitis”, “progesterone hypersensitivity”), “in vitro fertilization”, “pregnancy”, or “assisted reproduction”, respectively. In our comprehensive review of the literature, we included papers consisting of reports of women with APD that had either manifested itself during assisted conception or emerged in individuals who had undergone fertility treatments at any point prior to the onset of disease. We also collected data regarding reproductive success in people who had developed APD and possible treatment strategies for these patients. Considering the relative rarity of the disease, coupled with the relatively recent emergence of ARTs, our study encompasses a comprehensive range of original research articles with diverse research methodologies. Thus, we were able to include two retrospective studies, one observational prospective study, an observational case series, and three case reports. Our examination involved a total of 242 patients, with 215 having undergone assisted reproductive technologies (ARTs). Within this subgroup, 41 individuals were diagnosed with progesterone hypersensitivity, and data regarding reproductive outcomes were provided for 35 of these cases.

## 3. Results

In the selected studies, we identified 41 cases documenting progesterone hypersensitivity within assisted reproduction cycles. 

### 3.1. Reproductive History and Exposure Factors

All the cases detailed herein involved the administration of exogenous progesterone during an IVF cycle through various methods. Among the articles that reported the route of administration (35 cases), the most common approach for IVF treatment was intramuscular administration, at least as the initial preference in therapy. Intravaginal suppositories, gels, or creams were the initial types of progesterone supplementation in only three cases and were considered as the second or third choice in three cases (Table 1) [12,20,21,22]. Curiously, one patient, who had been previously exposed to OCs, tolerated induction therapy with medroxyprogesterone and leuprolide but started developing symptoms after intravaginal progesterone gel had been introduced [12].

The majority of the outlined cases lacked a history of progesterone hypersensitivity before undergoing IVF treatment (Table 2). This observation lends support to the hypothesis that sensitizing phenomena tend to occur during IVF procedures. Notable exceptions include one case where APD developed after the use of oral contraceptives (OCs); another case where APD manifested itself after pregnancy, although OCs had been previously administered in that case too; and a third case reporting endogenously triggered APD before the initiation of IVF [12,23,24]. Moreover, it is noteworthy to emphasize that 11 (26.8%) patients in our review had prior exogenous progesterone exposure, a much smaller percentage than the one within the study conducted by Foer et al. where more than half (58%) of the identified cases of APD were associated with exogenous triggers. In addition to IVF, emergency contraception, intra-uterine devices (IUDs), progesterone injections for a threatened abortion or uterine bleeding, and, more significantly, OCs have all been signaled as catalysts for APD (Table 2) [22,23,24]. 

Moreover, a significant portion of the cases pertained to individuals who were not undergoing their initial assisted reproduction cycle (Table 2). Notably, three patients reported repetitive unsuccessful IVF attempts, seven patients had an undetailed history of assisted reproductive technologies (ARTs), and one patient encountered recurrent unsuccessful intrauterine insemination [20,21,24]. Among the aforementioned patients, the majority opted for in vitro fertilization (IVF) due to difficulties in conceiving or infertility. For cases with detailed IVF history data, it was observed that the majority had undergone an average of three cycles of IVF previously, as seen in Table 2. However, Sood et al. did not find any statistically significant association between urticaria appearance and a history of ARTs [25].

**Table 1:** Demographic data and clinical manifestations of APD.

Author, Year	Type of Study	Studied Population	History of PH *	Route of Administration/Type of P *	Dermatological Manifestations	Symptom Onset Timeline
Hill, 2013 [20]	Case report	N = one 26 year-old (yr) female upon 7 weeks of gestation after IVF	NO	Intramuscular (IM), then	Urticaria on thighs, abdomen, and buttocks	2 h
				intravaginal suppositories and cream	Vaginal irritation with burning, pruritus, and blistering lesions	Delayed
Gupta, 2018 [21]	Case report	N = one 27 yr female	NO	Injectable natural micronized P	Burning and pain at injection site, fever, and breathlessness	2 h
				Aqueous P	Burning and pain at injection site, fever, and breathlessness	N/A
				Intravaginal gel and oral capsules	Vaginal blisters, fever, and breathlessness	N/A
Jenkins, 2008 [12]	Case report	N = one 43 yr female with APD	YES	Intravaginal gel	Pruritic, pink edematous plaques and macules on her upper thighs, axillae, and buttocks	2 days
Jo, 2019 [22]	Retrospective study	N = nine women with exogenous APD N1 = three had APD after IVF	NO	Intravaginal	Urticaria, itching, dyspnea, and hypotension	9 days
			NO	Intravaginal	Erythema and itching	1 h
			NO	IM	Erythema, urticaria, itching, and fever	6 days
Prieto-Garcia, 2011 [24]	Case series report	N = six women with APD N1 = three women with IVF-related HP	NO	IM	Urticarial rash on her abdomen and periorbital swelling	N/A
			YES	IM	Maculopapular rash on her face and abdomen	N/A
			NO	IM	Diffuse urticaria; lip, periorbital, and hand angioedema; dyspnea; chest tightness; lightheadedness; nausea; abdominal colic; diarrhea; diaphoresis; and presyncope	N/A
Foer, 2016 [23]	Retrospective study	N = 24 patients with APD N1 = six (25%) women who had undergone IVF	One case	N/A	Dermatitis, urticaria, angioedema, asthma, and anaphylaxis	N/A
Sood, 2018 [25]	Prospective study	N = 200 patients undergoing IVF	NO	IM	N1 = 26 cases of urticaria after P injection	N/A

* Progesterone hypersensitivity (PH), progesterone (P).

### 3.2. Clinical Aspects

From a clinical standpoint, urticaria emerged as the most prevalent manifestation of APD, followed by maculopapular rashes, erythema, pruritus, and edema, with periorbital swelling being notably recounted. Furthermore, systemic symptoms, including dyspnea, fever, angioedema, and ultimately anaphylaxis, were reported in the documented cases; however, as comorbidities were not often described, it was not possible to identify factors predicting severe reactions. In patients with intravaginal administration of progesterone pruritus, burning sensations and blistering were described (Table 1) [20,21]. 

Among the reports providing a temporal sequence from progesterone administration to the onset of symptoms, three cases indicated immediate hypersensitivity. However, systemic symptoms were observed in a single patient (Table 1) [12,20,21,22]. Conversely, it is noteworthy that progesterone was administered intravaginally in two of the three instances characterized by the development of APD as a delayed hypersensitivity reaction [20,22]. This observation suggests a potential association between intravaginal administration and an increased susceptibility to delayed reactions. 

Just as Foer and colleagues discussed, most people with secondary progesterone hypersensitivity do not experience persistent urticaria after IVF. However, our study revealed one case where chronic urticaria developed and another case where the person had a previous history of APD and started experiencing rashes again after delivery (Table 2) [22,24].

**Table 2:** ART history, case management, and outcomes.

Author, Year	Type of Study	Studied Population	P * Exposure and Obstetrical History	Treatment	Reproductive Outcomes
Hill, 2013 [20]	Case report	N = one 26 yr female upon 7 weeks of gestation after IVF	Recurrent IVF * treatments	Thirteen-step, rapid desensitization protocol with IM PIO * Target dose = 50 mg/day	Successful pregnancy No APD either during pregnancy or after delivery
Gupta, 2018 [21]	Case report	N = one 27 yr female	One spontaneous abortion at 6 weeks Three cycles of unsuccessful intrauterine insemination	Modified natural cycle (MNC)	Successful pregnancy
Jenkins 2008 [12]	Case report	N = one 43 yr female with APD	OC	High-potency topical corticosteroids	Three spontaneous abortions before a successful pregnancy
Jo, 2019 [22]	Retrospective study	N = nine women with exogenous APD N1 = three underwent P therapy for ART	Exposure to exogenous progesterone (the one in question not specified)	Antihistamines and systemic corticosteroids	N/A
				Antihistamines Desensitization protocol using IM P Target dose = 50 mg twice daily No premedication	N/A
				Antihistamines and systemic corticosteroids	Chronic urticaria
Prieto-Garcia, 2011 [24]	Case series report	N = six women with APD N1 = three women with IVF-related APD	Three cycles of IVF	Desensitization with intravaginal suppositories Target dose = 100 mg Premedication with prednisone	Successful pregnancy after fourth cycle of desensitization and IVF, with two previous miscarriages No APD either during pregnancy or after delivery
			OC exposure and one pregnancy	Desensitization protocol with intravaginal suppositories Target dose = 100 mg Premedication with prednisone	Successful pregnancy after two IVF treatments preceded by P-desensitization protocol. No APD during pregnancy. After delivery, she had the previous catamenial perioral rash
			Three cycles of IVF	Desensitization protocol with intravaginal suppositories Target dose = 100 mg Premedication with montelukast	Successful pregnancy after one cycle
Foer, 2016 [23]	Retrospective study	N = 24 patients with APD N1 = 6 (25%) women underwent IVF	N = 5/6 patients had APD only after exogenous P exposure (the one in question not specified)	Three patients underwent Rapid IM PIO desensitization Target dose = 50–75 mg daily, depending on IVF protocol	Two successful pregnancies
Sood, 2018 [25]	Prospective study	N = 200 patients undergoing IVF	N = seven urticaria patients had an ART history	N = 25 (92.6%) urticaria patients received oral antihistamines N = 2 urticaria patients received a short course of systemic corticosteroids	There was no significant association between urticaria and the outcome of IVF

* Progesterone (P), in vitro fertilization (IVF), progesterone in oil (PIO), oral contraceptives (OCs).

### 3.3. Treatment and Reproductive Outcome

Within the reported cases, a noteworthy observation pertains to the utilization of desensitization therapy for eight patients to enhance tolerance to in vitro fertilization (IVF) and support the intended pregnancy (Table 2). Three of these individuals followed a desensitization protocol involving intravaginal suppositories. In this protocol, the initial dose was progressively increased to reach 100 mg, the target dose for inducing ovulation and implantation. At the same time, these patients received premedication with prednisone or montelukast. Achieving reproductive success in this cohort required undergoing between one and four cycles of desensitization and in vitro fertilization (IVF). It is noteworthy to emphasize the absence of reported instances of failure to conceive within this group [24].

Among the five cases employing diverse intramuscular progesterone desensitization protocols, reproductive outcomes were documented in four instances. Three patients underwent rapid intramuscular progesterone desensitization, with progesterone suspended in sesame oil. These patients were maintained on a daily dosage ranging from 50 to 75 mg as per their IVF protocol. Notably, all patients tolerated the IVF process, and two pregnancies had occured by the time of the article’s publication [23]. Moreover, another patient followed a 13-step, rapid desensitization protocol that also utilized intramuscular progesterone in oil (PIO), with a target dose of 50 mg daily. This approach facilitated the completion of the initial IVF protocol, and the patient continued an additional five weeks of progesterone therapy for luteal phase support, resulting in a sustained pregnancy without further dermatological complications [20]. When the fertility outcome was not available, the documented desensitization procedure was conducted following a protocol utilizing intramuscular progesterone, with a target dose of 50 mg administered twice daily [22].

Another unique approach to APD was showcased by Gupta et al., who utilized the modified natural cycle (MNC) strategy. A follicle was induced through ovulation stimulation using tamoxifen and human menopausal gonadotropin. Ovulation itself was triggered by human chorionic gonadotropin (hCG), and the ensuing natural progesterone originated from the corpus luteum [21] This approach sought to avoid the administration of exogenous progesterone due to the patient’s exclusive hypersensitivity to exogenous progesterone. Consequently, a successful pregnancy had been achieved by the time of the article’s submission.

In contrast, a case with preexisting APD that was exclusively treated with topical corticosteroids witnessed three spontaneous abortions before achieving a successful term delivery, an occurrence that might be linked to the APD flares that complicated each first trimester [12]. Nonetheless, the extensive investigation conducted by Sood in 2018 concluded that there was no substantial correlation between urticaria and the outcome of in vitro fertilization, mentioning that patients were administered oral antihistaminics [25].

Conversely, the primary method employed by the overwhelming majority of patients for symptom control was the use of oral antihistamines. Additionally, the utilization of topical corticosteroids was reported. Four patients experiencing more systemic symptoms were administered systemic corticosteroids and one required epinephrine following intravaginal progesterone administration.

## 4. Discussion

### 4.1. Revisiting Progesterone in Fertility

Natural progesterone plays a vital role in essential physiological processes such as the menstrual cycle, implantation, and the maintenance of a pregnancy. It has been extensively utilized in the treatment of various gynecological conditions, including dysfunctional uterine bleeding, amenorrhea, and luteal phase deficiency, as well as in assisted reproductive technologies [26]. Referred to as “the pregnancy hormone”, progesterone is indispensable both before and during pregnancy, playing a pivotal role in its maintenance through various mechanisms. These include the modulation of the maternal immune response, the suppression of the inflammatory response, the reduction of uterine contractility, the enhancement of uteroplacental circulation, and the support of the luteal phase [27,28]. The luteal phase is defined as the duration from ovulation to the occurrence of pregnancy or the resumption of menses approximately two weeks later [29]. In a typical luteal phase, hormonal production peaks four days after ovulation and persists for about a week before declining in anticipation of the next menstruation. Following ovulation, granulosa cells undergo luteinization, which is influenced by the luteinizing hormone (LH), and the resulting corpus luteum relies on regular LH stimulation for adequate progesterone production [30]. In the event of pregnancy, the corpus luteum is sustained by human chorionic gonadotropin (hCG). The corpus luteum’s output is crucial in supporting early pregnancy; hence, a deficiency in the corpus luteum function is linked to both implantation failure and miscarriage [31]. 

Progesterone serves as a potent suppressor of the inflammatory response, particularly in the female reproductive tract where estrogen and progesterone modulate immune regulation [32]. Progesterone inhibits inflammation by promoting growth factor production, disrupting cytokine activity, and enhancing cellular repair pathways [33]. Its immunosuppressive role is crucial for pregnancy success, influencing various process stages. Progesterone suppresses pro-inflammatory responses and stimulates anti-inflammatory ones in the oviduct, creating an environment tolerant to foreign bodies like sperm and facilitating sperm viability. This immune tolerance may also pave the way for the development of fertilized embryos. Optimal estrogen signaling in the oviduct and progesterone action ensure a proper inflammatory balance during the embryo’s presence [34]. Throughout pregnancy, progesterone promotes the expansion and differentiation of regulatory T cells systemically and at the maternal–fetal interface, dampening the cytotoxic activity of natural killer (NK) cells [35]. Progesterone induces the production of progesterone-induced immunomodulatory proteins (PIBFs) by lymphocytes, which locally suppress the immune response at the fetal–maternal interface [34]. Additionally, progesterone polarizes circulating and tissue-resident immune cells toward an anti-inflammatory phenotype, downregulating the pro-inflammatory mediator release. It also regulates cellular immune processes in the cervix [35]. These progesterone-driven immunological changes collectively create a homeostatic state, ensuring a successful pregnancy by suppressing the inflammatory response and supporting maternal tolerance of the embryo/fetus.

As discussed previously, exposure to external progesterone can lead to the development of specific IgE antibodies for progestogens. This is supported by the frequent occurrence of immediate hypersensitivity and is also indicated by positive skin tests for progesterone. Upon subsequent exposure to progesterone, individuals may exhibit reactions caused by cross-linking these antibodies [36]. These IgE antibodies disrupt progesterone’s physiological functions, thereby diminishing its normal anti-inflammatory functions. This can pose additional challenges to conception and maintaining pregnancy as a decline in maternal tolerance of the embryo/fetus may occur.

The remarkable success rates of in vitro fertilization (IVF), now reaching up to 56%, contribute to the continuous increase of live birth rates over the last three decades and make it feasible for the technology to be used extensively in the future [37,38]. In an artificial cycle designed for endometrial preparation, the use of exogenous estrogen serves to inhibit follicular growth. Consequently, in the absence of a corpus luteum, supplemental exogenous progesterone becomes necessary to initiate and sustain the secretory endometrium, thereby facilitating pregnancy. Multiple studies, including those conducted by Bjurstrom, have demonstrated an improved live birth rate with the supplementation of progesterone during the luteal phase in frozen embryo transfer (FET) cycles [39]. Luteal-phase support (LPS) is a standard practice following embryo transfer in both fresh and frozen in vitro fertilization (IVF) cycles. LPS involves the administration of medications, primarily progesterone, to support implantation and pregnancy [40]. As hCG use may be associated with a higher risk of ovarian hyperstimulation syndrome (OHSS), progesterone remains the preferred agent of choice for luteal support [41]. Progesterone has been shown to facilitate the invasion of extravillous trophoblasts in the decidua by preventing apoptosis of these trophoblasts, affirming its rational use for luteal-phase support [42,43].

Therefore, in assisted reproductive cycles, supplementation with progesterone is indispensable post-ovum pick-up, and the effective management of progesterone hypersensitivity remains a notable challenge [23]. However, in a person who develops APD, any progesterone supplementation protocol might have nefarious results. A report by Kuruvilla et al. identified recurrent miscarriages as a complication of administering progesterone during the first trimester of pregnancy due to low hormone levels. The patient in question, who was prescribed vaginal progesterone for low hormone levels during her first pregnancy, experienced urticarial eruptions and thereupon suffered a miscarriage. In a subsequent pregnancy, a similar progesterone regimen resulted in an eczematous patch on her hand and another miscarriage. This suggests that progesterone may have the opposite of the desired effect when administered to an individual with suspected APD, potentially inducing miscarriages due to APD flare-ups [44].

Progesterone is available in various forms, including intramuscular, oral, vaginal, and subcutaneous formulations. Despite ensuring patient compliance, oral administration has drawbacks, such as poor bioavailability and rapid metabolism, that lead to plasma concentration variability. In assisted reproductive technologies (ARTs), concerns arise during pregnancy, including the production of interfering metabolites during liver passage and discrepancies between progesterone blood levels and the effect on endometrial histology in controlled stimulated cycles [27]. Conversely, supraphysiologic plasma levels were achieved following intramuscular administration, approximately seven times higher than those observed after vaginal administration; therefore, this reaffirms that intramuscular administration is the route that results in the highest blood levels [27]. However, over time, there has been an increasing preference for vaginal progesterone over intramuscular administration for luteal phase support [40,45]. This shift is influenced by recent findings indicating that vaginal progesterone supplementation results in significantly higher rates of implantation, delivery, and live births compared with intramuscular progesterone injection, whereby the latter is associated with a higher early abortion rate than vaginal progesterone [46]. Conversely, an alternative study suggests that supplementing vaginal progesterone with intramuscular administration appears to decrease the miscarriage rate and improve the live birth rate after oocyte donations [47].

### 4.2. What Is Known about Progesterone Hypersensitivity

Intolerance to sex steroid hormones has been previously observed, leading to a range of clinical symptoms such as dermatitis, premenstrual syndrome, dysmenorrhea, headache, arthralgia, asthma, rhinitis, acne, pruritus, mastalgia, and bullous erythema multiforme. Individuals experiencing these conditions may have a history of the cyclic or hormone-dependent symptoms of the above-mentioned maladies [48]. Harmful immune responses and hypersensitivity reactions are more common in xenoestrogenic environments and situations where progesterone levels are high. Both these conditions are met during assisted pregnancy and, consequentially, their incidence may increase during this delicate time [49].

It is also noteworthy that two of the patients analyzed in this research had a history of endometriosis as the onset of autoimmune progesterone dermatitis in a patient with endometriosis is rarely described in the literature [21,24]. This observation merits attention and further research, especially considering that, similarly to women with progesterone hypersensitivity, those with endometriosis experience immune imbalances that can impact implantation processes, pregnancy outcomes, and IVF outcomes [50,51,52,53]. Additionally, investigating the rarity of APD associated with endometriosis could offer a compelling research avenue. This scarcity may be attributed to the fact that the concentration of progesterone required to induce hypersensitivity phenomena such as dermatitis is infrequent in women with baseline progesterone deficiency. Consequently, women with higher baseline progesterone levels, such as women with polycystic ovary syndrome, may be more prone to such manifestations, although this hypothesis requires further research too.

While sex hormone sensitivity and autoimmune progesterone dermatitis are acknowledged concepts, the precise mechanism behind this hypersensitivity has not been fully elucidated [8,13,24]. Numerous theories have been suggested, with the most widely accepted one being an immunoglobulin E (IgE)-mediated response to progesterone, which is commonly substantiated through progesterone skin test positivity; however, a novel direct progesterone sIgE ELISA assay has been formulated to aid in the diagnosis [54,55,56]. Nevertheless, the mechanism through which patients initially become sensitive to progesterones remains unclear. Sensitization can occur through exogenous progesterone exposure, generating progesterone-specific IgE antibodies that cross-react with the rising endogenous progesterone levels during the luteal phase of the menstrual cycle [24,57]. Yet, endogenous progesterone hypersensitivity can develop without prior exposure to progesterone, implying steroid cross-sensitivity as a potential alternative sensitization mechanism [58,59]. Notably, not all patients with progesterone hypersensitivity (APD) displayed clinical features indicative of IgE-mediated reactions; some cases demonstrated delayed hypersensitivity responses [60,61]. This phenomenon can be explained by the modulation of Th2 through G-protein receptors or the activation of a progesterone membrane receptor α on CD8+ cells [62,63] An alternative explanation suggests the involvement of an immune complex-mediated mechanism [59]. Although our review focuses on APD urticaria being triggered through exogenous hormone administration, there are reported cases of chronic urticaria being successfully treated with contraceptives such as cyproterone acetate and ethinylestradiol; therefore, progesterone cannot be perceived as a risk factor for urticaria [57].

Classical catamenial APD manifests itself 3–10 days before menses, with symptoms disappearing shortly after. The clinical manifestations are highly polymorphous, though there is a consensus among most studies indicating a notably higher prevalence of urticaria [8,15,16]. Other frequently encountered manifestations include vesiculobullous Eeruptions, erythema multiforme, eczema, maculopapular rashes, and angioedema [8,15,16]. Rare cases presenting petechiae, purpura, necrotic migratory erythema, or dyshidrosiform lesions have also been described [64,65,66]. Furthermore, the clinical manifestations can evolve over time, as evidenced through a notable case report. In this instance, a woman with autoimmune progesterone dermatitis (APD) who initially experienced urticaria and dyspnea later developed a fixed drug eruption-like erythema after undergoing a progesterone challenge test [61]. Patients typically experience the same symptoms with each flare, with pruritus being the most frequent one, which is followed by respiratory distress. Edema of the extremities was reported in individuals with urticarial lesions, while some patients complained of soreness from burning and pain at the site of the cutaneous lesions [8]. The heterogeneous presentation of symptoms poses challenges to the diagnostic process; therefore, the diagnosis of APD relies on the correlation of symptoms with the menstrual cycle that include, respectively, synchronization with exogenous progesterone administration or peaks in endogenous progesterone, such as those occurring in early pregnancy or due to multiple pregnancies. A novel classification system for APD based on its initial trigger, such as endogenous or exogenous progestogens, has been introduced as a practical diagnostic tool [23]. By focusing on exposures rather than symptoms, this classification system may also help with early diagnosis as it draws attention to cases where the etiology might be endogenous, such as in cases triggered by pregnancy, or multifactorial, such as a combination of the previous use of OCs and pregnancy [67,68,69].

Confirmation of suspected autoimmune progesterone dermatitis (APD) involves conducting an intradermal skin test with progesterone, which is typically administered during the follicular phase of the menstrual cycle [55,70,71]. While an immediate urticarial reaction may be observed within 20–30 min in some cases, a more common occurrence is a delayed hypersensitivity reaction manifesting itself after 48–72 h. On account of the low sensitivity and specificity of progesterone skin testing for APD, allergists could also contemplate performing a progestogen challenge [72]. This is also feasible when physicians are faced with a lack of progesterone in an aqueous solution for intradermal allergen testing; the intravaginal progesterone provocation test is a viable method for substantiating progesterone sensitivity [73,74]. This has not come as a surprise as symptom appearance upon intravaginal progesterone administration in patients with autoimmune progesterone dermatitis during infertility treatment has also been previously reported [12]. Histopathologic findings in APD are generally nonspecific and often align with the morphology of the lesions [55,75].

### 4.3. Progesterone Hypersensitivity and Its Impact on Fertility

Pharmacological interventions employed through ARTs to enhance the likelihood of successful pregnancy may lead to diverse cutaneous manifestations. For instance, clomiphene citrate, utilized for follicle stimulation, can induce allergic reactions in the form of a pruritic morbilliform rash and thrombocytopenia [76]. Additionally, urine-derived gonadotropin hypersensitivity, rashes, injection site skin reactions to human menopausal gonadotropin (HMG), and the follicle-stimulating hormone (FSH) have been reported [77,78].

As previously discussed, the use of exogenous and/or supraphysiologic dosages of hormonal therapy for contraception or fertility treatments is associated with an elevated risk of developing dermatological disorders, particularly urticaria lesions. The utilization of progesterone during IVF has been associated with an escalation in a plethora of dermatologic manifestations. The intramuscular injections of synthetic progesterone for three weeks during her initial cycle of in vitro fertilization–embryo transfer were notably associated with photosensitivity localized in a vitiliginous facial lesion in a specific case [79]. Moreover, in a comprehensive case–control study, after a comparative analysis of single pregnancies, it was established that the incidence of pruritic urticarial papules and plaques of pregnancy (PUPPP) was significantly higher in individuals undergoing in vitro fertilization [80]. Notably, among IVF patients with PUPPP, there was a notable increase in both the duration of luteal-phase support with progesterone and the frequency of multiple pregnancies compared with IVF patients without PUPPP (*p* < 0.001 for both factors) [80]. Furthermore, PUPPP has been previously linked with increased progesterone receptor immunoreactivity and multiple pregnancy-induced high progesterone levels [81,82]. This prompts consideration of a potential association between PUPPP, the most prevalent gestational dermatosis, and sustained exogenous progesterone exposure, much like that seen with APD [83]. However, definitive confirmation of this link awaits further comprehensive research.

Autoimmune progesterone dermatitis has also been marked as a critical factor linked to infertility; an association between primary unexplained recurrent pregnancy loss and skin test reactivity to female sex hormones has been proven [84,85]. The most commonly cited reactions to progesterone suggest either a type one or a type four hypersensitivity reaction. As overall inflammation increases and the body combats the supplementation, the proven anti-inflammatory properties of progesterone are suppressed, leading to a sudden inefficacy of progesterone in its role of sustaining a pregnancy. This observation may have implications for clinical practice, suggesting a need for a more thorough anamnesis that focuses on symptoms and signs that may have previously emerged during the menstrual cycle’s luteal phase or during exogenous hormone exposure. This approach aims to minimize the risk of inducing miscarriages through standard progesterone supplementation techniques. In a study involving 29 women experiencing recurrent pregnancy loss due to an unknown cause, researchers conducted intracutaneous skin testing for estrogen and progesterone hypersensitivity. The majority of these women exhibited positive dermal test results, a phenomenon tjat was not observed in a control group. Subsequently, the researchers reported 16 successful pregnancies in women with recurrent miscarriages and positive skin tests for sex hormones after undergoing intradermal desensitization with estrogen and progesterone [85]. One subsequent study also claims that obstetric outcomes may be improved through desensitization. In this particular study, it was reported that 65% of patients with a history of habitual abortion experienced marked premenstrual symptoms, and the vast majority of them tested positive for either estradiol or progesterone, or they tested positive for both. Following a desensitization protocol, most of the patients reported a long-term and stable reduction in severe premenstrual syndrome (PMS), and 61% of them achieved live births in subsequent pregnancies [86].

There is an even broader range of methods through which APD has been documented to impact procreation. That being the case, the administration of 17a-Hydroxyprogesterone caproate (17P) therapy, commonly used to prevent preterm births during pregnancy, has been associated with the induction of iatrogenic APD [87]. Furthermore, vaginal progesterone administered due to a short cervix and a history of preterm delivery has been reported to induce delayed cutaneous hypersensitivity reactions [88]. Another noteworthy case of APD involves APD onset due to THE heightened sensitivity to endogenous progesterone manifesting itself a few months after a medical abortion induced using mifepristone and misoprostol tablets. Additionally, there have also been reports of the onset of APD following a spontaneous abortion resolved through uterine curettage, whereby the hypersensitivity was thus triggered by endogenous progesterone variations [89]. These reports underscore the importance of exercising caution whenever intervening in progesterone homeostasis, whether by administering exogenous progesterone or using progesterone antagonists such as mifepristone [90]. However, it is crucial to differentiate progesterone hypersensitivity from progesterone-vehicle hypersensitivity, whcih is a situation that has been previously documented particularly in cases involving progesterone-in-oil administration. In such instances, patients exhibited severe dyspnea and tachypnea due to the sesame oil component [91,92]. Patients experiencing hypersensitivity reactions to progesterone in sesame oil injections, including those with eosinophilic pneumonia, are advised to switch to a progesterone preparation with an alternative vehicle, such as peanut oil [91,93,94]. Moreover, advanced formulations of progesterone, which exhibit promising results in preclinical studies and limited clinical trials, present potential alternatives. However, despite their potential benefits for patients intolerant to traditional progesterone in oil formulations, dedicated research over the past few decades has not successfully translated these novel progesterone delivery systems into clinical applications [95].

Therefore, it is a predictable outcome that APD significantly impacts the success rates of IVF due to the difficulty in tolerating the supraphysiological doses of progesterone administered during IVF procedures [23]. Women experiencing catamenial symptoms, especially those who intend to undergo IVF, should undergo assessment for APD. This underscores the importance of understanding and addressing this dermatological condition in the context of fertility treatments.

### 4.4. Treatment

The most conservative and cautious therapeutic strategy when managing classical APD involves the use of oral H1 and H2 antihistamines in conjunction with topical and systemic corticosteroids to alleviate symptoms. The sole curative intervention entails the suppression of ovulation and endogenous progesterone production, which is achievable through the administration of combined oral contraceptives with both estrogen and progestin; however, complete resolution is seldom achieved [96]. It is advisable to utilize combined anovulatory drugs instead of isolated estrogen therapy because the latter involves administering high doses, a practice currently discouraged due to potential side effects. On the other hand, a paroxysmal reaction to a combined hormonal contraceptive vaginal ring (etonogestrel 0.120 mg and ethinyl estradiol 0.015 mg) has been reported. In the case mentioned above, the patient developed urticaria and generalized arthralgias less than 24 h after insertion [97]. In refractory cases, alternatives such as oral tamoxifen, an anti-estrogen agent, and danazol function by disrupting pituitary–hypothalamic regulation, thereby suppressing ovulation. Similarly, GnRH analogs exhibit favorable therapeutic outcomes, although their usage is limited due to cost considerations and the potential induction of premature menopause [96]. Regardless of age and the previous use of exogenous progesterones, bilateral oophorectomy has been reported to be a reliable treatment, especially for patients with severe symptoms such as Stevens–Johnson mimicking lesions or mucosal erythema multiforme [98,99,100].

Another potential approach involves desensitization to progesterone, a strategy examined in the cases outlined in our review, as detailed earlier. Desensitization emerges as a viable therapeutic option for patients who desire to preserve their fertility and who need progesterone therapy for other afflictions such as uterine bleeding [24]. Desensitization can be accomplished through protocols that employ incremental progesterone doses administered through various routes, including oral, intramuscular, or intravaginal administration [23,24].

This approach is particularly applicable to women undergoing infertility treatments, offering a means to tolerate fertility interventions while effectively managing symptoms, exactly as proven earlier [23]. Some authors cited herein underlined the successful process of desensitizing patients undergoing IVF by using vaginal suppositories with progesterone [24]. Incorporating premedication may have enhanced the procedure’s safety. Specifically, maculopapular rashes were treated with steroids, while hives and bronchospasms were prevented through montelukast administration. Furthermore, desensitization protocols involving intramuscular progesterone in oil (PIO) have been used in response to iatrogenic APD cases attributed to synthetic progesterone use during in vitro fertilization [20]. Moreover, as discussed previously, endogenous progesterone may also serve as an alternative to endometrial preparation in FET procedures in cases of exogenous APD in women undergoing IVF, exactly as Gupta et al. proved by utilizing the MNC strategy [21].

This research paper’s limitations stem from a relatively small pool of cases given that progesterone hypersensitivity is rare. Furthermore, focusing on its association with in vitro fertilization (IVF) narrowed the scope even more, potentially limiting the generalizability of the results. Hence, additional research is warranted. Conversely, in the literature concerning APD, endogenous progesterone, exogenous progesterone, and progestins are often collectively referred to as “progesterone”. Despite this grouping, these compounds differ on a molecular level, suggesting potential variations in their capacity to induce APD.

## 5. Conclusions

The utilization of in vitro fertilization (IVF) has witnessed a notable increase that is primarily attributed to delayed childbearing, and this upward trajectory is anticipated to persist. The paramount concern lies in ensuring the safety and efficacy of IVF treatments, particularly concerning progesterone usage. Our study supports the hypothesis that substantial exposure to elevated levels of exogenous progesterone during ARTs poses the risk of inducing hypersensitivity, prompting consideration of APD in instances of uncommon skin eruptions during fertility therapies. Nonetheless, prompt identification from reproductive endocrinologists and gynecologists, in particular, is essential to prevent diagnostic setbacks and failed IVF cycles as a result of progesterone intolerance. Desensitization emerges as the sole treatment option for women with APD seeking to preserve fertility, especially those undergoing infertility treatments who must continue progesterone therapy in order to obtain viable pregnancies. Even so, the exploration of progesterone hypersensitivity in the context of exogenous progesterone use, especially during ARTs, necessitates more extensive prospective studies. These studies should also prioritize comprehensive medical histories and thorough hormonal evaluations to precisely quantify risk factors associated with developing APD during IVF treatments. Ultimately, given the escalating administration of progesterone for infertility treatment, dermatologists, obstetricians, and allergologists must acquaint themselves with the rare entity of APD, its iatrogenic origins, and the available therapeutic interventions to ensure the continued accessibility and safety of fertility treatments for all women.

## Data Availability

Data sharing not applicable.
